# Aspects of Shared Decision Making in a Cognitive-Educational Intervention for Family Members of Persons Coping With Severe Mental Illness

**DOI:** 10.3389/fpsyt.2021.681118

**Published:** 2021-07-20

**Authors:** Penina Weiss, Dorit Redlich-Amirav, Sara Daass-Iraqi, Noami Hadas-Lidor

**Affiliations:** ^1^Occupational Therapy Department, Rabin Medical Center, Petach Tikva, Israel; ^2^National School of Mental Health Rehabilitation, Ono College, Kiryat Ono, Israel; ^3^Department of Occupational Therapy, Tel Aviv University, Tel Aviv, Israel

**Keywords:** family caregivers, dynamic cognitive intervention, shared decision making, mental health, Keshet

## Abstract

**Background:** Partnerships and family inclusion are embedded in mental health policies. Shared Decision Making (SDM) is as an effective health communication model designed to facilitate service users and providers engagement in reaching jointly decisions concerning interventions. Keshet is a 15 bi-weekly academic course for family members of people with mental illnesses that enhances positive family cognitive communication skills.

**Purpose:** To exhibit how SDM is inherently expressed in Keshet.

**Method:** We conducted a secondary analysis of previous Keshet evaluation studies and course protocols that focused on revealing SDM use.

**Results:** SDM was found to be a prominent feature in Keshet interventions in both the structure of the course as well as the process and procedures. Following participation in the program, making decisions jointly was found to be a prominent feature.

**Conclusions:** Interventions such as Keshet that include an SDM approach can contribute to the integration of academic, professional and “lived experience” within a shared perspective, thus promoting an enhanced equality- based SDM model that benefits individuals as well as mental health systems.

## Introduction

Family caregiving serves as the bedrock upon which health care systems tend to depend. However, caregivers face physical as well as emotional repercussions, due to the challenges they experience in the caregiver role, particularly in the mental health field (1).

The length of active caregiving by parents who have a daughter or son with mental illness often extends for many years (2, 3). During this long-time span, parents have to cope with a variety of challenges, including those of caregiving as well as from the additional accumulation of other life stressors that potentially all lead to experiencing psychological distress, due to their own health problems and psycho-social situations (1, 4). These caregiving and other life stressors may affect family caregivers in multiple ways, including increasing their experience of caregiving burden, elevating the risk of depression, and diminishing feelings of closeness to their family member who is coping with mental illness (5).

Maintaining family caregiver's health and well-being has been identified as a preventive public health promotion objective (6, 7). As families play a central role within the caregiving context, it is important to create and sustain equal and balanced partnerships with family members so they can provide specialized care while maintaining their own health, well-being, and resilience (1).

Since 2001, the Israel Ministry of Health is engaged in an accelerated process to develop community based psychiatric rehabilitation and recovery services as part of its implementation of the “Rehabilitation in the Community of Persons with a Psychiatric Disability law” (2000) (8). As part of this development, families of people coping with severe mental illness (SMI), are identified as a population that require specific and targeted needs. They are also perceived as a means to achieve effective recovery goals and outcomes. The Keshet program, described below, was developed as part of this process.

## Keshet (Rainbow in Hebrew)

Keshet is a didactic program held in academic settings intended primarily for family members of people coping with mental illnesses. The course provides tools for communication based on awareness and a meta-cognitive analysis of cognitive components within dialogues that take place within and outside the family. Keshet focuses on teaching parents/family members about cognition and thinking processes, as well as mediation. During the course human interactions are turned into a source of learning. It is based on a number of theoretical entities which include:

Theory of Structural Cognitive Modifiability (SCM) which means that cognitive structural changes are possible at any age and in any health status (9). This is based on the concept of brain plasticity and modifiability. This theory further explains that these structural cognitive changes occur by using Mediated Learning Experiences (MLE) an interventional approach that addresses communication, by which the mediator adjusts, filters, and enables processes and changes in a way that the learner will understand and achieve higher cognitive abilities (9–11). Employing MLE principles into the health field in general and particularly for family members of mental illnesses, is based on Dynamic Cognitive Intervention (DCI) developed by Hadas Lidor (12). Being taught these principles provide family members with more choice and control over various situations (12, 13).

The DCI within the mental health Recovery process, is an approach that emphasizes and supports an individual's ability to live with the illness and beyond it (14).

Choice and control over management decisions are important elements during the recovery process. Given the uncertainties involved, these elements are often associated with inherent tensions between the person, the family and professionals. Decisional conflict is also a central element in SDM process (15). The family member or the clinicians would identify communicative elements of SDM such as describing pros and cons of various options. In the Keshet course, choice and control are related to, not just as a part of Recovery, but as cognitive elements, that can be addressed as such in the discourse, as well as in the understanding, of a particular situation. Using DCI methods, family members can achieve joint control over a situation by opening various possibilities to choose from while respecting the choices of the family member as well (16). These conflicts about choice and control are central to SDM approaches and thus there are areas of overlap between Keshet and SDM. One of the purposes of Keshet is the transfer of DCI principles beyond the course into everyday life.

Feuerstein's SCM is based on the belief that every person has the potential to achieve cognitive development if he/she is exposed to supporting elements, such as Mediated Learning Experiences (MLE) within an environment that provides opportunities for active growth. The DCI approach, derived from the SCM theory, is specifically intended to enhance the therapeutic-based relationships in health-related fields with a direct emphasis on emotional issues and the way they affect cognitive development (12, 13).

DCI views the client as an equal partner in the therapeutic process. In this approach, not only do therapists work together with clients to select methods and goals, but they also convey to clients the central concepts of cognition, the steps involved in cognitive development and processes, and the clinical reasoning behind intervention techniques. Clients are exposed to the ways the cognitive communication skills based on mediation can enhance learning, adaptability, and recovery. This attitude leads to the partnership of everyone involved in the therapeutic process (16). Thus, in Keshet, parents are introduced to concepts and strategies usually used solely by clinicians.

This sharing of professional tools during Keshet, can be viewed as a strategy directed to promote SDM. Shared Decision Making is defined as an effective health communication model designed to facilitate patient engagement in treatment decision making. Engagement in decision making fosters communication skills by encouraging open dialogue with focus on empathy, trust and partnership (17–19). In its essence, after ensuring that the client is informed about his/her rights and options, SDM provides a space for trust, reciprocity, and mutual respect of relevant knowledge in decision making. Participating in SDM increases active participation and involvement of users in their care by eliciting an interactive and collaborative process between them and others (20, 21). Shared Decision Making (SDM) focuses on the centrality of experiential knowledge, alongside scientific knowledge (19, 22). SDM is aimed at reaching an agreed solution but agreeing to disagree is acceptable. A collaborative approach not only benefit user's treatment but also encourages them to become equal partners with professionals (23). SDM has been recognized as an effective health communication model designed to facilitate patient engagement primarily in mental health treatment decision making (24). Keshet and SDM both provide evidence to effectively contribute to personal recovery and decision making in psychiatric rehabilitation settings (1, 23, 24).

## Purpose

This paper describes a secondary analysis of Keshet components from inception to program evaluation post participation, with the purpose of highlighting elements related to SDM.

## Methods

Using secondary analysis is an integral part of research development, which provides a different set of skills to bear on the data. A secondary analysis of previous Keshet evaluation studies was used, in order to generate valuable practical insights and derive new additional interpretations and conclusions.

Keshet is a course designed as an intervention to fill the void concerning parents of adult children with mental illness- whom in this role spend much more time than professionals with family members coping with mental health illnesses, but are equipped with less tools than professionals for effective coping with everyday life challenges. The course is led a jointly by a professional and a family member who had participated previously in Keshet (25, 26). Central themes that Keshet addresses promote transfer of knowledge from professionals to families by exposure of participants to the way cognitive communication skills, based on mediation, can enhance learning, adaptability, and recovery in a way that promotes active involvement.

The central components taught together by a family member and a professional in Keshet, and which are included in meaningful mediated cognitive based communication according to both the SCM theory, MLE, and the DCI approach are:

1. *Intentionality and reciprocity*. The mediator's responsibility in any purposeful interaction is to ensure intent is clear to the recipient. Reciprocity is making sure that the idea, thought, or request was understood precisely, by the recipient, even though it is possible that the recipient did not necessarily agree with the expressed ideas. The structuring of communication based on these elements promotes feelings of trust and engagement.
2. *Transcendence*. An interaction that provides mediated learning must be also directed toward transcending the immediate needs or concerns of the recipient by venturing beyond the here and now, in space and time. Transcendence is the ability to make generalizations. Participants learn to use new communication strategies that include SDM approach and this is also transferred to their own personal relationships and interactions.
3. *Mediation of meaning*. This deals mainly with the energetic dimensions (increasing motivation) of the interaction (i.e., with why things happen or are done). It raises the individual's awareness and understanding and makes explicit the implicit reasons and motivations for doing things. Mediation of meaning focuses on the interaction of the individual with other people and aims to increase his or her ability to make choices (27, 28). Raising awareness of participants to the importance of relating to meaning (i.e., what/because I understand what matters to you, it matters to me) helps to create a trust building relationship directed at empathy and mutuality.
4. *Mediation of competence*. This parameter deals with the way the mediator helps the individual feel a sense of competence and ability, in relation to him- or herself and to the task s/he undertakes. Learning how to direct an exact sense of competence is an enabling experience which leads to a more profound emotional sense of belief in oneself and in others, which in turn develops into a basis for the sharing of ideas and thoughts.
5. *Sharing*. Sharing behavior implies the need of the individual to share his/her feelings, thoughts and experiences with another person. Loneliness and social exclusion are characteristic of many populations with disabilities. Sharing, which is a way to overcome this setback, has two aspects: sharing one's world, difficulties, and successes with someone else; as well as doing and experiencing things together such as brainstorming, decision making, playing, and traveling.

A central method used in Keshet, are MILEs (Meaningful Interactional Life Episode), which are authentic verbal interactions that are experienced between at least two people, who then write up and submit the dialogue. The purpose of the MILEs is to use authentic personal experiences in creating connections to theoretical concepts taught. Documenting episodes and their analyses serve a number of purposes. They support and connect theoretical concepts and terminology with authentic personal experiences. As theoretical terms unfold, participants are encouraged to return to the written episodes and explore how the new parameters of mediation are expressed or missed within the MILEs. The MILEs are read jointly by Keshet moderators who together determine the key components to address in each particular MILE. Family member moderators input is invaluable as their lived experiences add an empathetic perspective to the understanding of the MILE and lead to a more sensitive response to the participant who handed it in. All MILEs are returned to participants who wrote them with commentary written jointly by instructors, linking between the personal dialogue and the theoretical and practical structures being taught.

**Institutional Review Board** (IRB) approval was obtained for each of the original studies performed. As the current paper utilized a secondary analysis of previous data, ethical board approval was not required. The primary research assessed various aspects of intervention evaluation, such as changes in attitudes, problem solving, and communication abilities using quantitative and qualitative measures both prior to and following the course. In the present paper, we re-examined the studies with a focus on SDM criteria.

## Results

The Results section describes the way SDM is reflected within Keshet at all stages of course evolvement- inception, content, and process. Furthermore, previous studies that were readdressed from an SDM perspective during the secondary analysis, are presented.

### Course Inception

Hadas-Lidor and Weiss (25) outlined the major principles used in inception stage that reflect a SDM approach:

a. Before initiating the course, focus groups that included family members of mental health service users and professionals (occupational therapists) with expertise in mental health rehabilitation were held, aimed at partnering jointly to define course purpose, need, content, and structure. Family members' personal lived experiences were the basis that helped establish which components were to be included in Keshet.
b. The theoretical base for Keshet was based on partnership and collaboration, including lived experiences of both carers and service users as integral components, creating partnerships based on a non-judgmental stance (Recovery) and the mediation of Sharing.
c. Two versions of the course manual were developed; one for the professionals and users leading the course, and a parallel one for family caregiver participants which includes home assignments, thus reflecting SDM features of involvement, interactivity and collaboration (16).
d. Partnering with parents and other family members in the course leadership and mentoring, enabling an authentic and relevant learning process for all. Joint professional and family member mentors bi-weekly meeting was established, both before and during the intervention, in order to contribute and facilitate their full partnership.

Creating partnership and collaboration within Keshet were special changes and adaptations that were made in the course. Weiss et al. (29) describe the changes that were made to ensure compatibility for a specific population which required cultural adaptation, namely the ultra-orthodox Jewish community in Israel (26). This community is characterized by being secluded and viewing of modernity as a threat to religious beliefs. In order to create the same type of partnering as with other Keshet participants, this particular population required special adaptations to ensure the same comfort zone, level of involvement, and ability to transfer learning from course into their natural surroundings. Rabbis were consulted and changes were made in course material accordingly. For example, wording of material was changed, more references to religious belief, and foreign language expressions not commonly used within religious communities were removed from course Power Point presentations. This sharing of perspectives and changes formed better partnerships within the groups later on (30). This is based on the concept of Knowledge Translation (KT), a term that represents relating valued research findings to the clinical field. This concept was applied to the importance of promoting transference of knowledge used by professionals to families - conveying to participants' knowledge of central concepts of cognition, cognitive development and processes and the understanding of the clinical reasoning behind intervention techniques. That in turn enables sharing and can be viewed as a benchmark that later on promotes SDM, as family members gain a better and more holistic understanding of knowledge and strategies relevant to them and their service user family member. This became one of the central aspects of the program.

### Course Structure

The Keshet course involves teaching methodology that promotes SDM.

1. Joint teaching by two course leaders- The interaction between the leaders becomes a role model of communication techniques being taught. This demonstration of an interactive relationship based on MLE and Recovery concepts is used and mirrored through the course as an example of sharing, empathy and partnership (28). Joint reading and writing of responses to MILES as well as analysis of MILES within the course meetings together with participants include teaching of strategies and tools that relate to SDM (see example below in Table 1: MILE analysis).
3. Follow up- in order to encourage continued participation, connection, and involvement between group meeting, both professional and family member group leaders maintain contact- by phone or online. These interactions promote feelings of caring, trust, openness, and empathy among participants.

**Table 1:** MILE analysis.

**Text**	**MLE/DCI**	**SDM**	**Recovery**
We sat together and thought. Then we made a list together	Intention and reciprocity	Developing trust Collaborative task	Peer support, Respect
We focused on “…what is important to us … [and] what is most important to her…”	Meaning	Invitation for open communication and shared information	Person-centered, Strength-based
“We decided we would do an initial evaluation [of schools] according to the criteria (she decided about), and then we would think together again.”	Competency	Following her preferences Increases autonomy and involvement of service users in their care	Hope, Responsibility, Non-linear
Her and our preferences were mutually respected (a music track for her and full graduation for us)	Intention and reciprocity	Mutual respect Reaching a shared decision based on mutual respect and trust	Respect

### Course Content

Often, although not all family members participate in the courses, participants share with us (31) that they share with their family (spouse/children/service users) the course manual and material, thus creating a joint family learning experience, that in turn considerably improves family communication and decision making.

All five aspects of MLE described above, are achieved in Keshet by involvement, collaboration and partnership which are the essence of the SDM approach. These aspects, enhance active involvement, hope and resilience of family members who in turn aid the development of these communication strategies in relationships with meaningful others, whether with service users, professionals, or other family members (1, 32).

### An Example to Understand Key Programmatic Elements

These MLE components can be viewed in the SDM approach as described below:

1. Two people conversing in room each have meaningful knowledge that is equal in value and relevant to discussion- **Reciprocity, Sharing, Meaning**
2. Within the conversation there is certainty that the client is informed about their rights, ideas, and options- **Intention, Transference**
3. Ensure that the person I am conversing with has the ability to negotiate with me- **Intention and reciprocity**
4. Ability to converse- **Competence**
5. The outcome may go in either direction, is created within the conversation and is not known in advance- **Reciprocity, Sharing**

Decisional conflict is defined as personal uncertainty about which option to choose.

As mentioned above, instruction in Keshet is enhanced by the use of MILEs. Here is an example of a MILE handed in by a mother toward the end of a KESHET course.

“During the Keshet course, our daughter who copes with an anxiety disorder was to start school again. She refused to return to the previous school and we searched for a new educational framework together. In order to decide, we sat together and thought about what was important to her in school- we made a list.

We, as parents, emphasized that it is important to find a school that provides full matriculation exams. Then, we thought about how we would get the information about whether what is important to her and what is important to us as parents takes place in various schools. She only wanted to attend a school that provides everything that is important to her, particularly music. We decided we would do an initial evaluation according to the criteria, and then we would think together again. It turned out that not every school has all the things, and there are differences between the schools. We presented them to her and thought together what was most important to her among the criteria. I remembered it was very important to her that there be a music track there. As mentioned, it was very important for us to have the possibility of full graduation. And it was this combination that brought her to the school she eventually attended.”

Originally this MILE was analyzed according to MLE/ DCI principles. However, this MILE can be also viewed as an SDM process the parents had developed following Keshet.

If we look carefully at the above MILE, one can identify active listening and respect, mutual trust, invitation to an open communication and information.

The MILE analysis in Table 1 highlights the MLE and SDM components/attributes, that make for effective communication, within a Recovery based approach.

A third aspect that can be viewed as an SDM within Keshet components is embedded in another exercises given to Keshet participants, where they are requested to think about things they want to change in their lives. More often than not, family caregivers provide answers relating to what they would change in their **childrens**' lives. This is done, without consulting or involving the children themselves in the process. Parents are always amazed while acknowledging that something is lacking in the way they determine goals they want to change. Keshet moderators (a professional and a family member) assist participants in understanding that changes can be brought about only if the change is accomplished within a partnership in which the feeling, thoughts, desires, and dreams of the family member experiencing mental ill health are taken into consideration and treated with respect and encouragement. Following this, participants are requested to once again do this exercise, but this time together with the service users. Following this process, the goals they want to change are transformed. For example, parents want a change in that their son/daughter should keep his/her room tidy and to maintain good personal hygiene. But when asking their children what they would like to change, the latter have altogether much grander ambitions, such as getting married, be able to play a musical instrument or be able to leave their parents' home and live on their own. The goals are jointly redefined, taking both intents into a common, joint one.

The changes participants in Keshet achieves, are nonetheless of the stance of the consumer regarding the degree of involvement the consumer expects of the family member. For example, A son of a family member in one course had not spoken with her for 6 years prior to the course. During the sessions of the course, with the skills she developed during Keshet, they started making joint written decisions that eventually led to face to face meetings and conversation.

### Studies on Keshet Evaluation

A previously published qualitative study about KESHET (31) analyzed 14 course protocols from three stages of two different Keshet groups, namely beginning, middle, and end of courses. The study focused on participants' attitude changes regarding faith in ability to change, empowerment, acceptance and empathy, and the ability to apply problem solving skills to everyday conflictual interactions. The study found a shift, from not believing change is possible and feelings of pity and self-helplessness, to trust building relationships, mutual respect, and value of each participants' knowledge as meaningful and equal. One father, who experienced helplessness since his son did nothing all day … following Keshet, started passively joining him while watching basketball games … went on to discuss the games with him, and afterwards gradually with the improved communication with his son, went on to engage with him in carpentry- planning and building a bench together. The study also explored the change from a passive to an active stance. One of the changes participants experienced was in their ability to understand that a index family member's choices must be honored. Changes participants experienced throughout the course- led to enabling increased freedom to make choices, even identification with index family members' choice. This move enables them to become less defensive, with improved acceptance of family member, by the end of the course.

Mental illness creates uncertainty as well as helplessness for all involved, namely for both family members and service users. Often, parents join the course with the purpose of achieving more control over their children's lives, a tendency which in itself leads to unbearable tension. Keshet strengthened the parents by helping them through improved communication and awareness to change the focus of control to a more joint effort. In turn, this move which creates sharing and honoring of their family members opinions ends up by enhancing their sense of control (25, 26).

The parents learned to include the index family member in the decision making during the process as an alternative to being controlling (32). Elazari et al. (31) also points to the actual verbal/use of language by parents that affect the partnering with their family members from general wording that does not bring about change (like “it is hard to connect” at the beginning), to specific wording that can be used as practical stepping stones for creating change (e.g., “My sharing with her” toward the end of the course).

In a study that addressed attitudes of parents regarding knowledge, beliefs, and action changes following participation in Keshet, participants attitude regarding inclusion of service users in decision making improved significantly following Keshet (33).

In another study based on both quantitative and qualitative methodology, participants found to be significantly more confident in their mastery of tools for coping with MILEs following the course in comparison with beginning of course (34). Three themes were under covered which are essential to the SDM process. (1) Keshet is an attempt to go beyond the despair and frustration to improved relationships with self, child, and the health system; (2) Keshet is a means to improve communication empowerment and feelings of competency and (3) The group leaders have a meaningful role and effect on learning and promoting recovery and change (35).

In a recent meeting of Keshet moderators and graduates, a follow up discussion targeting SDM was conducted. This was done, in order to directly inspect SDM use by Keshet participants prior to and post participation. Participants and professionals reported a change in SDM use within their family following their exposure to Keshet.

## Discussion

The purpose of this paper was to examine the place of SDM approach within the Keshet intervention, while initially acknowledging that SDM was not a theoretical entity introduced purposefully into the intervention. Furthermore, this secondary analysis serves the purpose of suggesting that the Keshet intervention can be used as a base for teaching and learning SDM communication strategies.

SDM is an approach that has previously been perceived as a pathway to aid effective and collaborative shared medical decision making, by service users together with professionals (21) and family members (36, 37). It is our belief that SDM can be regarded as a broader, therapeutic, as well as an interactional, effective communication approach. To validate this belief, the present study demonstrates ways SDM is inherently expressed in a cognitive communication intervention for family caregivers. With time, it became apparent that this cognitive communication intervention includes elements of SDM at all stages of the course development and throughout the course itself. As hopefully we head toward an era of personalized medicine that differs from the “one size fits all” approach, it is important to advance interventions that reflect partnerships and collaborations between professionals and users in a way that individuals have a voice that is heard, seen and related to. Keshet is unique in the way it manages to bring about changes (i.e., more sharing behaviors, giving more of a voice to the family service user) in the individual, although it can be defined as a psychoeducational group intervention, which is done primarily via the use of MILEs.

This paper applied a secondary analysis approach to broaden and deepen knowledge. SDM in Keshet can be seen in course inception process, course content, and process as well as in outcomes.

In recent years SDM has been proved beneficiary for service users, family members, and professionals, in terms of improved relationships and outcomes, primarily regarding health related decisions. Research findings highlight that SDM has a positive impact on reducing the length of hospitalizations, increased compliance and satisfaction with medical treatment (21, 25).

Keshet is an intervention based on a number of theories and approaches that promote improved communication and relationships. These are:

1. Feuerstein's SCM that postulates the belief in a person's ability to change and develop regardless of his/her diagnosis, etiology, and age.
2. MLE and environment- both provide the settings in which change can be set into motion.
3. Recovery- Concepts such as honor, respect, and making choices taken from the mental health recovery perspective are inherent in Keshet and have added value within a SDM based relationship.
4. Knowledge Translation (KT), defined as a dynamic and iterative process which includes synthesis, dissemination, exchange, and ethically-sound application of knowledge to improve health, provide more effective health services and products while strengthening the health care system (38, 39). In Keshet, principles of mediated communication lead to translated knowledge, intended to be used by family members in order to achieve SDM through enabling equality and equity in any meaningful relationship (40). The clinician or family member is responsible to ensure the client has all necessary information s/he needs to make informed choices in any life matter and in all interpersonal relationships.

In order for this to happen, the course developers had to reach the realization that the partnership between professionals and family members is the key pathway to improved communication, potential cognitive development and participation, and well-being. While the therapist comes and goes, family members are a stable entity in the service users lives and as such must have the tools to enable growth and development for all involved.

In terms of practical implications, it is apparent that Keshet can be used as an intervention that has the potential to support the development of the SDM capacity of course participants leading to improved interactions within healthcare institutions, as well as familial improved communication, participation, and well-being.

Hence, it is important to stress, that there are reciprocal ties between Keshet and SDM that provide added value and benefits, as the learning of the mediation language helps participants undercover and develop a more structured “language” based SDM set of skills.

Integrated descriptions of shared decision making exist, but many focus only on medical decision making. There is much to benefit from a broader approach, which takes SDM into everyday life situations.

## Conceptual or Methodological Constraints

As this study is primarily a secondary analysis, it is primarily descriptive with some qualitative components. Additionally, the sample size of the original study section was small. Therefore, it may have limited generalization capacity. It is important to provide evidence to the presence of SDM in Keshet via larger quantitative and controlled trials to attest the usefulness of Keshet as a tool to develop SDM strategies among participants.

## Future Directions

Future evidence-based research should be conducted with the purpose of addressing the use of SDM methodologically both prior to, and following participation, in Keshet. Likewise, it is equally important to continue evidence-based research to further establish Keshet as an SDM based intervention. To the best of our knowledge, this is the first time an intervention that was not originally developed from focusing on the SDM process, was analyzed according to SDM principles. Further studies might look at other interventions through an SDM scope to add an in-depth dimension to client centered care and well-being.

In the mental health, it may be important to expose service users and family members to the elements taught in the Keshet intervention. Holding courses for all members of the family can create a setting where DCI theory can be put into practice jointly by service users together with their family members.

## Author Contributions

PW, DR-A, and NH-L contributed to conception and design of the study, performed the secondary analysis, and wrote sections of the manuscript. SD-I organized the references and styling. All authors contributed to manuscript revision, read, and approved the submitted version.

## Conflict of Interest

The authors declare that the research was conducted in the absence of any commercial or financial relationships that could be construed as a potential conflict of interest.
